# Foliar Substrate Affects Cuticular Hydrocarbon Profiles and Intraspecific Aggression in the Leafcutter Ant *Atta sexdens*

**DOI:** 10.3390/insects6010141

**Published:** 2015-02-12

**Authors:** Lohan Valadares, Daniela Nascimento, Fabio S. Nascimento

**Affiliations:** Departamento de Biologia, Universidade de São Paulo, Avenida Bandeirantes, 3900 Ribeirão Preto, Brazil; E-Mails: dlndandara@gmail.com (D.N.); fsnascim@usp.br (F.S.N.)

**Keywords:** *Atta sexdens*, intraspecific recognition, cuticular hydrocarbons, foliar substrate

## Abstract

Cuticular hydrocarbons (CHCs) are traditionally considered to be one of the most important chemical cues used in the nestmate recognition process of social hymenopterans. However, it has been suggested that in the leafcutter ant genus *Atta*, it is not the CHCs, but the alarm pheromone that is involved in the nestmate recognition process. In this study we used a laboratory population of *Atta sexdens* to explore the association between their CHC profile variation and intraspecific aggression. In the first part of the experiment, four colonies were divided into two groups with distinct diets to stimulate differentiation of their CHC profiles. In the second part of the experiment, all colonies received the same diet to examine resemblance of chemical profiles. At the end of each part of the experiment we extracted the CHCs from workers. The results demonstrated that colonies that shared the same food resource had similar cuticular hydrocarbon profiles. Furthermore, colonies were significantly more aggressive towards conspecifics that used a different foliar substrate and consequently had greater differences in their cuticular chemical composition. This study suggests that the CHC profiles of *A. sexdens* can be affected by the foliar substrates used, and that the CHCs are used in the nestmate recognition process of this species.

## 1. Introduction

The cohesion of hymenopteran societies is strongly associated with the ability of their members to discriminate relatives and non-relatives. The inclusive fitness concept predicts the adaptive gain for the individual that gives up their own reproduction to promote the reproduction and survival of related individuals [1]. The benefits of such an intricate system directly depend on nestmate recognition, which also can be interpreted as the first defensive line for the colony and its resources [2]. The process of recognition usually follows three main components: production, perception and response [3,4]. The production process requires a visual or chemical component, or a group of cues used by individuals for recognition [5,6,7,8,9]. The process of perception occurs when an individual detects the cues or signals from another individual, comparing it with the preformed representation of the colonial odor [10,11]. The next process is a response to the detected signal, where reactions can range from ignoring the individual, through antennation up to extreme aggression, followed by the death of both checked individual and the resident one [6].

The body surface of the insect is known to be covered by a cuticle, of which outermost layer, the epicuticle, is composed of a mixture of lipids that contain hydrocarbons. The composition of the lipids may contain hydrocarbons. There is a general consensus that cuticular hydrocarbons (CHCs) play a major role in the recognition system, as they serve as recognition cues between two or more individuals [12]. However, Hernandéz and colleagues [13,14] have controversially suggested that in the leafcutter ant genus *Atta*, major recognition cues are compounds originating from mandibular glands, the alarm pheromone, discarding the role of CHCs.

In addition to genetic factors, environmental factors can affect the composition of CHCs, making the profile of these compounds subject to temporal changes according to seasonal variation, nesting substrate, food and the presence of symbionts and pathogens [15,16,17,18,19,20]. In the agricultural system of *Atta* leafcutter ants, both workers and their symbiotic fungus *Leucoagaricus gongylophorus* feed on the glucose generated by the degradation of foliar starch [21,22]. Based on the extensive processing of leaves by ant workers it can be hypothesized that the foliar substrate is one of the major environmental factors influencing CHC profiles of those ants. Consequently, this study aimed to improve the understanding of the role of CHCs in the recognition system of *Atta sexdens*, by investigating the association between their CHC profile variation and intraspecific aggression. The two specific objectives of this study were: (1) to investigate the temporal changes in the CHC composition in relationship with the type of foliar substrate being used and (2) to measure intraspecific aggression between colonies and associate it to their degree of CHC profile resemblance.

## 2. Experimental Section

### 2.1. Diet Groups

Four colonies were collected from locations that were at least 50 km apart, near the cities of Botucatu (Colony A1), Rio Claro (Colony A2 and Colony B2) and Ribeirão Preto (Colony B1) in São Paulo State, Brazil, during their initial phase (approximately 3 months old). They were kept in laboratory conditions until the age of two years, when the experiments were conducted. At that time, colonies A1 and B1 had approximately 5 L of fungus garden and colonies A2 and B2 had 2.5 L.

The controlled feeding experiment was conducted for 120 days. For the first 60 days (Phase I), colonies A1 and A2 (Group A) were fed exclusively *Rosa* sp. leaves and petals. At the same time, colonies B1 and B2 (Group B) were fed exclusively with *Lagerstroemia* sp. Through the next 60 days (Phase II), all colonies were fed exclusively with flowers and leaves of *Hibiscus* sp.

### 2.2. Behavioral Assays

At the end of each phase, 10 individuals from each colony, one individual at the time, were introduced to a foraging arena of a colony (a “test” colony) for 2 min, where the nestmates served as control. In total 160 subjects were introduced to four forging arenas (*n* = 40 each) in each phase of the study. Introductions were made on a rotation system in order to minimize stress in a colony. To avoid bias, the observer did not know the colony origin of the introduced individual. Preliminary tests using ink-painting techniques showed that this procedure of marking subjects was not appropriate for this study, because workers were able to easily identify marked subjects. Therefore, we decided to distinguish subjects introduced to forging arenas by removing their hindleg tarsus (the procedure did not affect the performance of the ants). All introduced test subjects were removed after the behavioral data had been collected. We assumed that the number of individuals in the larger colonies was greater so we controlled the number of workers in the foraging area to approximately 30 during the experiment.

**Figure 1:**
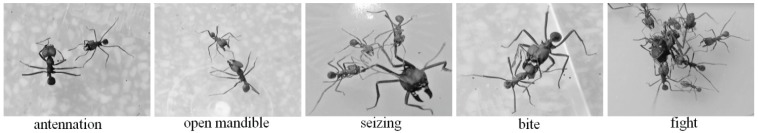
Behavioral acts registered during aggression assays using four *Atta sexdens* colonies.

The behavioral responses of resident workers towards an introduced subject were noted using an adapted version of a five-point scale [23]: 1—antennation; 2—opened mandibles; 3—seizing; 4—bites; and 5—fights (Figure 1).

Later, the aggression index (AI) was calculated using the following formula:

$$AI=\frac{\text{ total number of interactions scoring }3,\;4\text{ and }5}{\text{total number of all interactions}}$$

Possible A.I. scores range from 0 (where no aggressive interaction 3, 4 and 5 has occurred) to 1.0 (highest A.I. score). We compared the A.I from both phases using the Mann-Whitney *U* test. We then applied a generalized linear model (GLM) for all five behavioral categories with a with a binomial error structure used to reveal which factors affected recognition (acceptance or rejection). Phases and category of introduced ant (nestmate or non-nestmate) were analyzed as fixed factors in order to test variation in their rates of recognition. Donor colonies were assigned as random factors [24].

### 2.3. Chemical Analysis

Cuticular compounds were extracted from foragers at the end of each phase (*n* = 15 per colony) in 130 microliters of hexane for two minutes and analyzed in a combined gas chromatography-mass spectrometry GC-MS (Shimadzu, Tokyo, Japan, model QP2010 plus) equipped with silica capillary column and helium as carrier gas at 1 mL/min. The oven temperature was initially set to 150 °C, increasing 3 °C/min until it reached 280 °C (maintained for 10 minutes) and again 10 °C/min until reach 300 °C (maintained for 15 min). The data were analyzed with CG-MS solution for Windows (Shimadzu Corporation) and chemical compounds were identified based of their mass spectra by comparison with the Wiley and NIST Library database and with a standard solution of different synthetic hydrocarbons.

The identification of alkene double bound position was made through derivatization method of hexane extracts from 20 foragers with Dimethyl Disulphide (DMDS) [25]. The extract was dried with nitrogen and re-suspended in 200 microliters of hexane. Subsequently, 200 microliters of DMDS (Sigma-Aldrich, St. Louis, MO, United States) and 100 microliters of iodine solution (dissolved in diethyl ether, 6% p/v) were added. The vial was then purged with nitrogen, closed, and agitated at ambient temperature for 24 h. Thereafter, the mixture was diluted in hexane and 5% sodium thiosulfate solution, thereby extracting the organic phase, which was subsequently dried with sodium sulfate and analyzed in the GC-MS.

For chemical analysis data, multivariate analysis was conducted with a single factor PERMANOVA using 9999 permutations. Compounds that were present in less than eight individuals per group, as well as compounds contributing less than 0.5% to the total compounds were excluded from analysis. The data were standardized using root squared, and a Euclidean distance resemblance matrix was calculated on normalized data. Pairwise tests were made by comparing CHC profiles between colonies and phases. Canonical analysis was performed to classify the samples in their groups. These calculations were carried out using the statistical software Primer version 6 (Primer-e Ltd., Lutton, UK, 2009).

## 3. Results

### 3.1. Behavioral Assays

**Figure 2:**
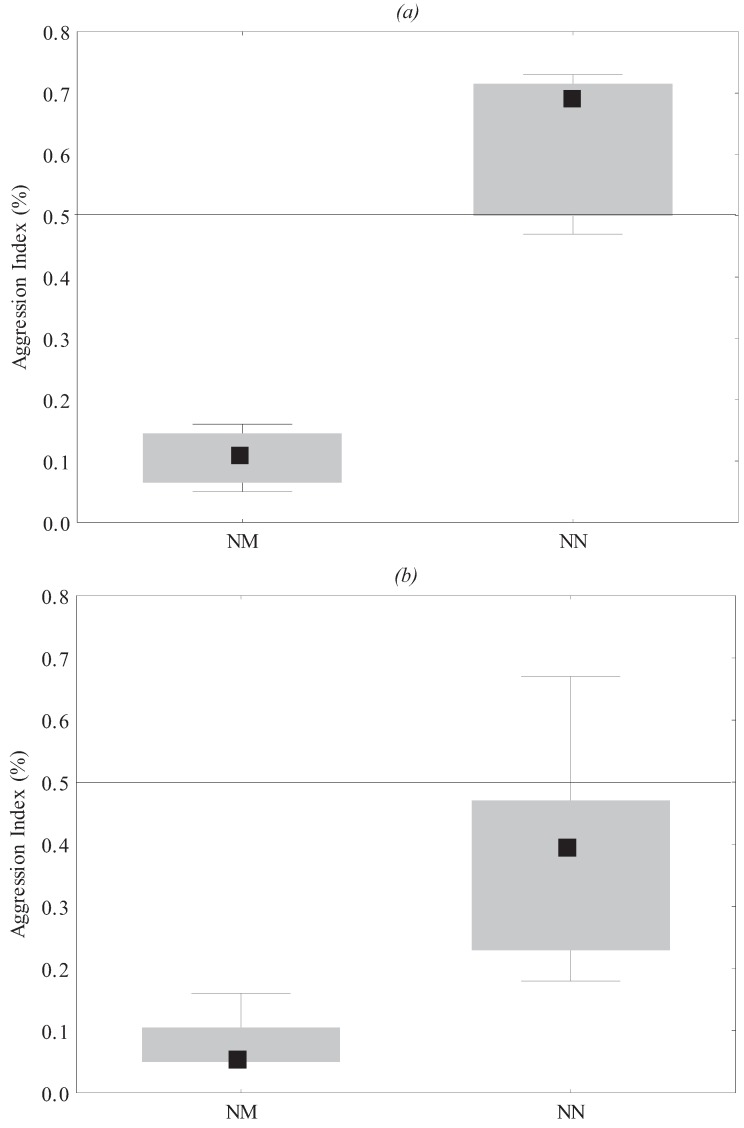
Frequency of aggression indexes between colonies from group-A* vs.* group-B at (**a**) Phase I and (**b**) Phase II. NM = nestmates; NN = non-nestmates. Black dots = mean; gray boxes = standard error; whiskers = standard deviation.

We found out no significance for the analysis involving A.I for all possible combinations across phase I and II (*U* = 81.5, *p* = 0.079). However, when we compared, only the A.I from Group-A* vs.* Group-B across both phases, was found to be significant (*U* = 5, *p* < 0.0045), indicating that the resident workers showed higher levels of aggression against conspecifics that used different foliar substrate. The nestmate introductions (control) compared to the non-nestmate ones demonstrated to be significantly different in each phase (*U* = 0, *p* < 0.0065 for each phase) but there were no significance when we compare only the controls between the phases (*U* = 5, *p* = 0.3528). The combinations involving colonies from group A* vs.* group B showed the highest level of aggression in both phases of experiment, but a salient decrease of the aggression had occurred in Phase II (Figure 2). When considering the interaction categories, we observed a significant difference in the frequency of open mandibles, bites and fights throughout the phases, with a higher frequency in Phase I (open mandibles GLM, *p* < 0.00007; bites GLM, *p* < 0.0026; fights GLM, *p* < 0.0259). In contrast, seizing did not show significant differences between the phases (GLM, *p* = 0.31) and antennation occurred in all introductions.

### 3.2. Chemical Analyses

**Figure 3:**
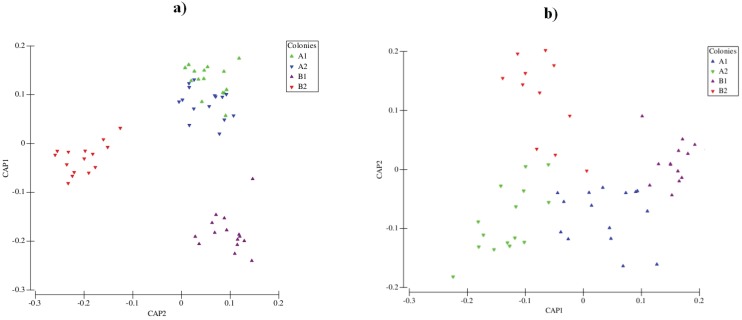
Result of canonical analysis of principle coordinates carried out with cuticular hydrocarbons of four *Atta sexdens* colonies at (**a**) Phase I and (**b**) Phase II.

Cuticular compounds of *A. sexdens* were mainly hydrocarbons classified as alkenes, alkanes, monoalkanes, dimethylalkanes and trimethylalkanes ranging from 19 to 40 carbon atoms (Table 1). The CHC profiles of colonies were significantly different overall (Pseudo-F = 14.563 *p <* 0.0001) and changed themselves from Phase I to the Phase II (Figure 3) according to the comparisons of the pairwise test for each colony (Col A1 *p <* 0.0001, Col A2 *p <* 0.0003, Col B1 *p <* 0.0001, Col B2 *p <* 0.0017). The calculation of Euclidean distance together with the pairwise test comparisons carried out with the combinations of the colony CHC profiles showed that Phase I colonies were chemically more different (Table 2).

**Table 1:** Mean and standard deviation of the relative proportions of cuticular hydrocarbons of four *Atta sexdens* colonies (A1, A2, B1 and B2) at both experimental phases (PI-I phase; PII phase-II).

Compounds	A1		A2		B1		B2	
	PI	PII	PI	PII	PI	PII	PI	PII
*Z*-9-C_19_	-	-	-	-	1.04 ± 1.83	-	-	-
*Z,Z*-C_32:2_	-	-	-	-	0.69 ± 1.24	-	-	-
*n*-C_25_	5 ± 1.64	6.32 ± 2.40	4.28 ± 1.65	5.97 ± 1.39	7.93 ± 2.28	6.25 ± 2.14	7.47 ± 228	6.32 ± 1.21
*n*-C_26_	-	0.54 ± 0.36	-	-	0.59 ± 0.44	-	0.78 ± 0.34	-
*n*-C_27_	10.5 ± 2.85	9.49 ± 2.48	10.33 ± 3.02	9.36 ± 2.20	17.0 ± 7.78	14.46 ± 2.57	10.92 ± 2.35	8.49 ± 1.93
*n*-C_29_	1.8 ± 0.68	0.62 ± 0.59	1.84 ± 0.88	1.23 ± 0.63	4.47 ± 3.77	4.14 ± 2.04	1.69 ± 0.7	0.92 ± 0.27
*a,b,c-*TriMeC_30_	-	-	-	-	0.61 ± 0.56	-	-	-
3,7,11-trimeC_30_	4.72 ± 1.17	-	2.87 ± 1.28	-	7.71 ± 2.46	-	3.72 ± 1.55	-
*n*-C_31_	1.03 ± 0.23	4.65 ± 1.52	0.58 ± 0.36	4.17 ± 1.57	-	4.41 ± 0.96	-	3.92 ± 1.14
9-meC_31_	0.76 ± 0.35	-	-	0.69 ± 0.41	0.85 ± 0.72	-	0.6 ± 0.55	-
3,5-dimeC_31_	3.8 ± 1.41	3.22 ± 1.07	2.26 ± 1.09	2.8 ± 1.05	4.87 ± 1.86	2.48 ± 0.88	3.13 ± 1.5	3.37 ± 0.96
Unknown	8.36 ± 1.8	3.88 ± 0.67	5.67 ± 1.79	5.84 ± 1.74	8.65 ± 3.53	3.6 ± 0.78	4.92 ± 2.04	5.39 ± 1.57
3,7,11-trimeC_32_	13.5 ± 3.03	8.08 ± 1.71	10.15 ± 3.26	8.28 ± 2.74	14.30 ± 4.76	6.97 ± 1.41	6.94 ± 2.5	8.12 ± 1.85
*n*-C_33_	1.08 ± 0.26	-	0.56 ± 0.39	-	-	-	-	-
4,8,12-TriMeC_33_	7.07 ± 1.44	3 ± 0.55	4.82 ± 1.56	4.02 ± 1.28	5.64 ± 2.87	2.55 ± 0.62	4.58 ± 1.91	5.08 ± 1.39
*a,b,c*-TriMeC_33_	6.12 ± 1.35	3.14 ± 0.75	4.08 ± 1.43	3.03 ± 1.12	4.84 ± 2.38	2.52 ± 0.61	3.43 ± 1.33	4.14 ± 1.29
Unknown	-	6.07 ± 3.82	-	7.99 ± 4.84	-	2.62 ± 1.75	-	4.55 ± 3.31
Unknown	1.08 ± 0.29	-	-	0.73 ± 0.30	0.54 ± 0.52	-	-	0.61 ± 0.36
*a,b,c*-TriMeC_34_	1.53 ± 0.57	0.58 ± 0.26	0.88 ± 0.61	1.1 ± 0.61	0.87 ± 0.66	-	0.84 ± 0.48	0.97 ± 0.25
3,7,11-trimeC_34_	5.54 ± 1.12	2.77 ± 0.57	3.68 ± 1.58	2.78 ± 1.36	4.11 ± 1.99	2.13 ± 0.47	2.78 ± 1.23	3.38 ± 1.01
Unknown	1.36 ± 1.38	-	1.34 ± 1.27	-	-	-	-	-
4,8,12-trimeC_35_	0.95 ± 0.33	-	-	-	-	-	0.71 ± 0.44	0.69 ± 0.49
*a,b,c*-TriMeC_35_	3.49 ± 2.2	5.64 ± 1.94	6.94 ± 3.21	4.31 ± 1.74	2.99 ± 2.15	7.01 ± 1.07	6.79 ± 2.89	4.44 ± 1.12
Unknown	0.5 ± 0.37	-	-	-	-	-	-	-
*a,b,c*-TriMeC_37_	13.54 ± 5.35	19.26 ± 3.98	24.33 ± 6.50	16.39 ± 4.69	8.97 ± 5.36	22.83 ± 3.28	23.7 ± 7.95	19.94 ± 3.10
*a,b,c*-TriMeC_39_	7.87 ± 3.24	22.03 ± 3.39	14.88 ± 4.41	20.34 ± 4.42	3.33 ± 2.34	18.04 ± 2.89	15.6 ± 6.27	19.63 ± 3.28
*a,b,c*-TriMeC_40_	-	-	0.51 ± 0.63	-	-	-	1.4 ± 1.66	-

In regards to the specificity of compounds in concentrations above 0.5% for a diet group or experimental phase, we found that the linear alkane *n-*C_33_ was only present in phase-I. In the first phase of the experiment, the colony A1 had as major compound the 3,7,11-TriMeC_32_, but in phase-II, the *a,b,c-*TriMeC_39_ compound was abundant. For colony B2 the compound *a,b,c-*TriMeC_37_ was majority in both phases as well as for colony A2 at phase I, which in the phase-II had the compound *a,b,c*-TriMeC_39_ as the major one. For colony B_1_ the linear alkane *n*-C_27_ was the major compound and in the Phase II it was *a,b,c*-TriMeC_37_ (Table 1).

**Table 2:** Euclidean distance and Pairwise tests carried out with the cuticular hydrocarbon profiles of *A. sexdens* colonies (A1, A1, B1 e B2) at the Phase I and II of the experiment.

Phase	Colony Combinations	Euclidean Distance	Pairwise Test (*p* value)
I	A1* vs.* A1	2.95	-
I	A1* vs.* A2	3.85	0.0001
I	A1* vs.* B1	4.48	0.0001
I	A1* vs.* B2	4.28	0.0008
I	A2* vs.* A2	2.8	-
I	A2* vs.* B1	5.25	0.0001
I	A2* vs.* B2	3.49	0.0554
I	B1* vs.* B1	3.97	-
I	B1* vs.* B2	5.21	0.0001
I	B2* vs.* B2	3.14	-
II	A1* vs.* A1	2.58	0.0608
II	A1* vs.* A2	2.94	0.0001
II	A1* vs.* B1	2.99	0.0033
II	A1* vs.* B2	2.69	-
II	A2* vs.* A2	2.61	-
II	A2* vs.* B1	3.37	0.0001
II	A2* vs.* B2	2.79	0.0439
II	B1* vs.* B1	1.83	-
II	B1* vs.* B2	3.05	0.0001
II	B2* vs.* B2	2.16	-

## 4. Discussion

This study demonstrates that foliar substrate is one of the environmental factors influencing the CHC profile of the leafcutter ant *Atta sexdens*. Importantly, this profile seems to be associated with recognition processes in this species, as colonies with greater differences in their chemical profiles showed highest levels of aggression. The same pattern was previously found for other two leafcutter ant species, *Acromyrmex echinatior* [25,26] and *Acromyrmex subterraneus* [27]. Our data combined with the previous works support the importance of CHCs in recognition mechanisms of leafcutter ants, contradicting the suggestion proposed by Hernández and colleagues where they discarded their importance [13,14]. Hernández* et al.* has been supported by Jaffé [28,29,30,31,32] who suggests that “non-advanced” ant species use cues provided by the environment, such as CHCs, or a blend of exocrine glands, while, species with “advanced” nestmate recognition mainly use an alarm pheromone.

Vander Meer and Morel in their review on nestmate recognition in ants [2] have already discussed the position of alarm pheromones in recognition by explaining that they are a result of recognition and not its cause. They argued that it is unlikely that alarm pheromones act as recognition cues because they must be spread over the individual’s entire body surface and in a proportion lower than the alarm response threshold, otherwise alarm behavior would occur constantly. Hernández* et al.* [13,14], rejected the role of CHC in recognition based on an experiment using extracts from the postpharyngeal gland (PPG) that did not elicit intraspecific aggression in *Atta* workers. Their PPG extracts were mainly linear hydrocarbons as well as branched ones, most of them found in the cuticle of the workers that they analyzed, and concluded that chemical structures such as hydrocarbons could not possibly be involved in discrimination between self and others in leafcutter ants.

It is true that hydrocarbons produced by epidermal cells can be transported through the haemolymph and to be stored in PPGs of ants, as has been shown specifically in *Cataglyphis niger* [33], however little is known about how hydrocarbons are transported and arranged on the cuticle of most insects [15]. Therefore, future studies involving the transport and arrangement of cuticular hydrocarbons in social insects are urgently required for a better understanding of the utility of these compounds as recognition cues. In particular for *Atta* leafcutting ants, studies approaching the direct influence of their mutualistic fungus *Leucoagaricus gongylophorus* and its hydrocarbons in the composition of *Atta sexdens* CHCs as well as the real influence of certain hydrocarbons structures must be conducted to better understand recognition systems and the chemical ecology of this ant.

## 5. Conclusions

In summary, our results demonstrate that for *A. sexdens*, the foliar substrate processed by the workers seems to affect the CHC profile, making the colony odor subject to temporal changes. Such changes are reflected in the nestmate recognition of this ant, as colonies that were fed on a different resource showed highest level of aggression compared to colonies that shared the same resource.
